# The Austrian Corona Panel Project: monitoring individual and societal dynamics amidst the COVID-19 crisis

**DOI:** 10.1057/s41304-020-00294-7

**Published:** 2020-10-22

**Authors:** Bernhard Kittel, Sylvia Kritzinger, Hajo Boomgaarden, Barbara Prainsack, Jakob-Moritz Eberl, Fabian Kalleitner, Noëlle S. Lebernegg, Julia Partheymüller, Carolina Plescia, David W. Schiestl, Lukas Schlogl

**Affiliations:** 1 Department of Economic Sociology, University of Vienna, Kolingasse 14-16, 1090 Vienna, Austria; 2 Department of Government, University of Vienna, Kolingasse 14-16, 1090 Vienna, Austria; 3 Department of Communication, University of Vienna, Kolingasse 14-16, 1090 Vienna, Austria; 4 Department of Political Science, University of Vienna, Universitätsstraße 7, 1010 Vienna, Austria; 5 Vienna Center for Electoral Research, University of Vienna, Kolingasse 14-16, 1090 Vienna, Austria

**Keywords:** Behaviour and attitudes, Coronavirus, COVID-19, Online panel survey, Pandemic response, Survey data, Social sciences

## Abstract

Systematic and openly accessible data are vital to the scientific understanding of the social, political, and economic consequences of the COVID-19 pandemic. This article introduces the Austrian Corona Panel Project (ACPP), which has generated a unique, publicly available data set from late March 2020 onwards. ACPP has been designed to capture the social, political, and economic impact of the COVID-19 crisis on the Austrian population on a weekly basis. The thematic scope of the study covers several core dimensions related to the individual and societal impact of the COVID-19 crisis. The panel survey has a sample size of approximately 1500 respondents per wave. It contains questions that are asked every week, complemented by domain-specific modules to explore specific topics in more detail. The article presents details on the data collection process, data quality, the potential for analysis, and the modalities of data access pertaining to the first ten waves of the study.

## Introduction

The COVID-19 crisis has fundamentally changed everyday life in Austria as well as in many other countries. Some people have been seriously ill. Some have experienced income or job losses, and those fortunate enough to work from home often struggle with combining housework and childcare. Students and their families have faced the various challenges related to home schooling. In sum, the Austrian population has experienced a period of rapid change. However, the challenges and implications for every person and family have been different. Given the severity of the COVID-19 crisis and its unprecedented disruption of many areas of social and economic life, it is vital to be able to systematically track and understand the consequences of such disruptions among the general population. Researchers from various areas need access to data in an open and free manner. This allows generating systematic scientific evidence and thereby contributes to the understanding of the crisis.

In this article, we present the publicly available data set of the first ten survey waves of the Austrian Corona Panel Project (ACPP), which has been a multidisciplinary effort of social scientists from the University of Vienna investigating how information, attitudes, and behaviours are distributed across the population, and how these develop in the course of the crisis. This unique Austrian data set^1^ spans the period between the end of March 2020, 2 weeks after the announcement of the general lockdown in Austria, and early June 2020, when the measures curtailing economic activity have been lifted in most sectors (excluding, in particular, culture and sports) and everyday life slowly went back to “normal”. Data were collected through a weekly online panel survey with a core set of standard questions (asked every week) and a variety of modules asked once or at longer intervals. The data thus allow to trace individual- and group-level reactions to the crisis and to the political and societal responses, as these evolved over time.

The aim of this article is to introduce the academic community to the data set by describing the research design, the method of data collection, and the basic structure of the data. In the following sections, we first describe the aims of the project, present the data, and discuss some methodological aspects of data collection. We then describe the themes covered in the various waves of the panel and illustrate selected research potentials with some descriptive findings. This discussion is followed by an explanation of data access and a few concluding remarks.

## Aims of the survey

Austria has responded early and with rather strict measures to the COVID-19 pandemic, which resulted in a flat infection curve but generated a near-to-complete economic lockdown for 10 weeks. The responses have brought about various dilemmas. For instance, in the early phase of the pandemic, the Austrian government prioritized infection control measures over any other considerations. This strong focus on public health aspects in the government’s and legislators’ responses, we argue, was important to contain the number of infections. At the same time, the focus on the threat of the virus temporarily pushed other concerns, such as routine medical care or psychological well-being, into the background.

After the first phase,^2^ it became visible that the crisis has already widened economic inequality and exposed new groups to economic hardship. Families have struggled with restrictions on movement and home schooling—if not with loss of income. The fear of infecting others has fostered practices of solidarity and, at the same time, concerns for people’s own health have deepened existing societal cleavages and created new ones. Large societal challenges, in particular climate change and forced migration, have been superseded by more immediate concerns raised by soaring unemployment and economic hardships. Overall, assessing the desirability of health measures solely against economic and employment objectives, or solely against democratic and civil rights, may lead to neglecting wider societal challenges and its interrelations. The aim of ACPP is, thus, to overcome the dichotomy between public health and economic considerations and to pay attention to the whole range of societal, economic, and political factors that can be affected by a crisis. We seek to contribute to a more encompassing picture of various areas of life during the COVID-19 crisis and investigate individual, societal, and political reactions and how they are related to each other.^3^

The project thus aims for a holistic representation of pandemic responses and their societal consequences by generating a data set that covers a broad range of aspects in a multitude of points in time, and allows to study the causal relationships and interlinkages between the various aspects at the individual and group level. The data allow systematically tracing consequences of the crisis that go beyond measures to control infection and to prevent the overburdening of healthcare systems. We are particularly attentive to non-intended consequences of measures and policies as they can burden those who are already disadvantaged, which in turn negatively affects their social and economic conditions. In view of the serious danger of a “syndemic pandemic” that affects people with worse health status more strongly than others (Bambra et al. 2020), the crisis might deepen societal cleavages and lead to political polarization across Europe and beyond. ACPP aims to contribute a multidisciplinary and integrated view on how different aspects of the handling of the COVID-19 crisis might reinforce or interact with existing inequalities.

Specifically, the project is designed to trace important interrelations between the pandemic and the response measures on the one hand, and different societal fields affected by the pandemic and the externalities of responses on the other hand. It seeks to analyse the interlocking impacts of the COVID-19 crisis on individual behaviour and social dynamics in different societal fields by answering, among others, the following core questions: How do people see the threats at a health and economic level—both for society at large as well as for the person individually? How do people feel about and react to the political measures taken? What do they think of the democratic and communicative challenges? How have different social groups been affected by the crisis and how do existing inequalities interact with the crisis’ impact on individuals and the society?

## Study design and data quality

For these aims, ACPP established an online access panel survey with a sample of 1500 respondents that is representative of the socio-demographic structure of the Austrian population. The panel design means that the same people are being interviewed repeatedly to track changes in a time of rapid change. Questionnaires contain a broad range of questions to capture individual experiences with the COVID-19 and the ensuing economic crisis, asking about respondents’ situations, perceptions, emotions, attitudes, preferences, and behaviours. A core set of questions is asked weekly to the same group of people. In addition, (alternating) modules explore certain dimensions in greater detail, yet at greater intervals. This enables us to track important trends in a granular manner in various domains of practice and policy and to focus on important specific questions in particular fields that arise in certain points during the crisis.

### Study design and fieldwork period

**Fig. 1:**
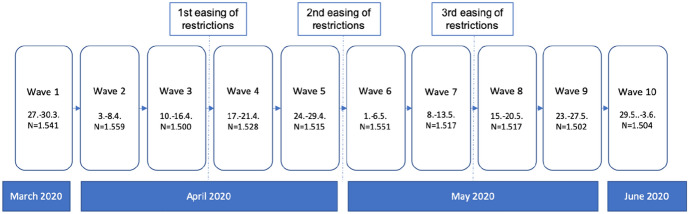
Schedule of waves

Data collection started on 27 March 2020, precisely 14 days after the announcement of the lockdown by the Austrian government on 13 March 2020 (which came into effect on 16 March 2020). The survey started exactly at the time when infection numbers peaked. The severe lockdown measures that were in effect during the first waves of our data collection were subsequently eased in several stages, beginning in the middle of April (see Fig. 1).

Over a period of 10 weeks, the same group of respondents was invited to participate in the weekly survey. Thanks to the short, weekly intervals, the survey is ideally suited to capture the rapidly unfolding societal and individual dynamics amidst the COVID-19 crisis and for a study of the dynamics of public opinion and behaviour during a period of intense crisis communication in a fine-grained way.

### Data quality: coverage, accuracy, and weights

Survey respondents were recruited from a pre-existing online access panel provided by Marketagent.com online research GmbH (Baden, Austria; certified under ISO 20252). Marketagent has a total of 129,500 registered panellists in Austria and routinely recruits panellists using various channels for off-line recruitment, which includes advertising on television, print media, radio, and billboards, to reduce bias towards heavy online users in its pool. Marketagent had proven to be a reliable data partner in previous academic projects such as the Austrian National Election Study (AUTNES) Online Panel Study 2017–2019 (Aichholzer et al. 2020).

To participate in the survey of the Austrian Corona Panel Project, respondents need to reside in Austria, be at least 14 years old, and have access to the internet (either via smartphone, tablet, or computer). Respondents from the access pool were selected using quota sampling and were invited based on quotas for the following key demographics: age, gender, age*gender, region (Bundesland), municipality size, and educational level.

To assess the accuracy of the raw and weighted data, Table 1 displays the deviations as measured by the root mean square error (RMSE; MacInnis et al. 2018) for all waves and across a number of demographic variables for which data from official statistics (Statistik Austria 2019) are available. Both the raw and the weighted data are very accurate, with an overall average deviation of about 3 percentage points for the raw data. In the raw data, the largest deviations can be found for educational groups. Further in-depth analysis reveals that these deviations are caused by the underrepresentation of respondents at the lowest level of education—a common non-response problem in most surveys. The second largest deviation is found for household size, with larger households (3 + persons) being somewhat overrepresented at the cost of two-person households. Most other deviations are quite small.

**Table 1:** Accuracy measures for the raw and weighted data

Variable	W1	W2	W3	W4	W5	W6	W7	W8	W9	W10	Post-stratification weights?
Gender (2 cat.)^a^	0.9	0.6	0.4	1.1	0.4	0.8	0.8	0.9	0.5	1.3	No
Age (6 cat.)^a^	3.0	2.2	2.3	2.3	2.3	2.6	2.2	2.3	2.1	2.2	No
Gender*Age (12 cat.)^a^	1.6	1.2	1.2	1.3	1.2	1.4	1.3	1.3	1.2	1.3	No
Education (5 cat.)^a^	7.0	7.1	7.3	7.7	7.4	7.3	7.5	7.4	7.7	7.8	No
Region (9 cat.)^a^	0.4	1.0	0.4	0.6	0.5	0.3	0.3	0.5	0.5	0.3	No
Household size (3 cat.)	4.4	4.1	4.3	4.4	4.0	4.3	4.4	3.8	4.7	4.4	No
Employment status (4 cat.)	2.0	1.7	1.6	1.8	2.0	2.1	1.9	1.7	1.6	1.7	No
Migration background (2 cat.)	3.7	3.3	2.7	2.7	2.1	2.2	2.6	2.8	1.6	1.8	No
Overall (43 cat.)	3.2	3.0	3.0	3.2	3.0	3.1	3.1	3.1	3.2	3.2	No
Gender (2 cat.)^a^	0.0	0.0	0.0	0.0	0.0	0.0	0.0	0.0	0.0	0.0	Yes
Age (6 cat.)^a^	0.1	0.1	0.1	0.1	0.1	0.1	0.1	0.1	0.1	0.0	Yes
Gender*Age (12 cat.)^a^	0.0	0.0	0.0	0.0	0.0	0.0	0.0	0.0	0.0	0.0	Yes
Education (5 cat.)^a^	0.0	0.0	0.0	0.0	0.0	0.0	0.0	0.0	0.0	0.0	Yes
Region (9 cat.)^a^	0.0	0.0	0.0	0.0	0.0	0.0	0.0	0.0	0.0	0.0	Yes
Household size (3 cat.)	0.0	0.0	0.0	0.0	0.0	0.0	0.0	0.0	0.0	0.0	Yes
Employment status (4 cat.)	0.0	0.0	0.0	0.0	0.0	0.0	0.0	0.0	0.0	0.0	Yes
Migration background (2 cat.)	0.0	0.0	0.0	0.0	0.0	0.0	0.0	0.0	0.0	0.0	Yes
Overall (43 cat.)	0.0	0.0	0.0	0.0	0.0	0.0	0.0	0.0	0.0	0.0	Yes

Entries are RMSE values (percentage points) calculated based on Table 3 in “Appendix”

^a^Demographics with categories (partially) affected by quotas

The demographic post-stratification weights (W*_WEIGHTD) available in the data set remove the deviations from the population targets completely. Tiny deviations remain for age but those are within the margin of tolerance (0.1) of the iterative-proportional-fitting weighting algorithm (Bergmann 2011) that was used to calculate the weights.

All in all, the survey estimates mirror the target distributions of the Austrian population fairly closely both for quota and other demographic variables. Data users should always keep in mind, though, that data from online access panel surveys with non-probability sampling needs to be interpreted with great care as self-selection, mode, undercoverage, and non-response can still cause biases when studying specific outcomes, especially, when factors are related to the likelihood of respondents entering the panel (Kohler et al. 2019; Cornesse et al. 2020).

### Data quality: response rates, retention, and patterns of panel attrition

The initial response rate in wave 1 was 35.2%: Out of 4381 invited panellists, 1541 interviews were completed. This response rate can be considered unusually high. For comparison, the initial response rate in wave 1 of the AUTNES Online Panel Study, 2017–2019, was 9.0%, recruiting from the same access pool of respondents. Thus, the study profited from the fact that many people had to stay at home and were more willing to spend their time by answering survey questions (Dillman et al. 2014; Keusch 2015).

**Fig. 2:**
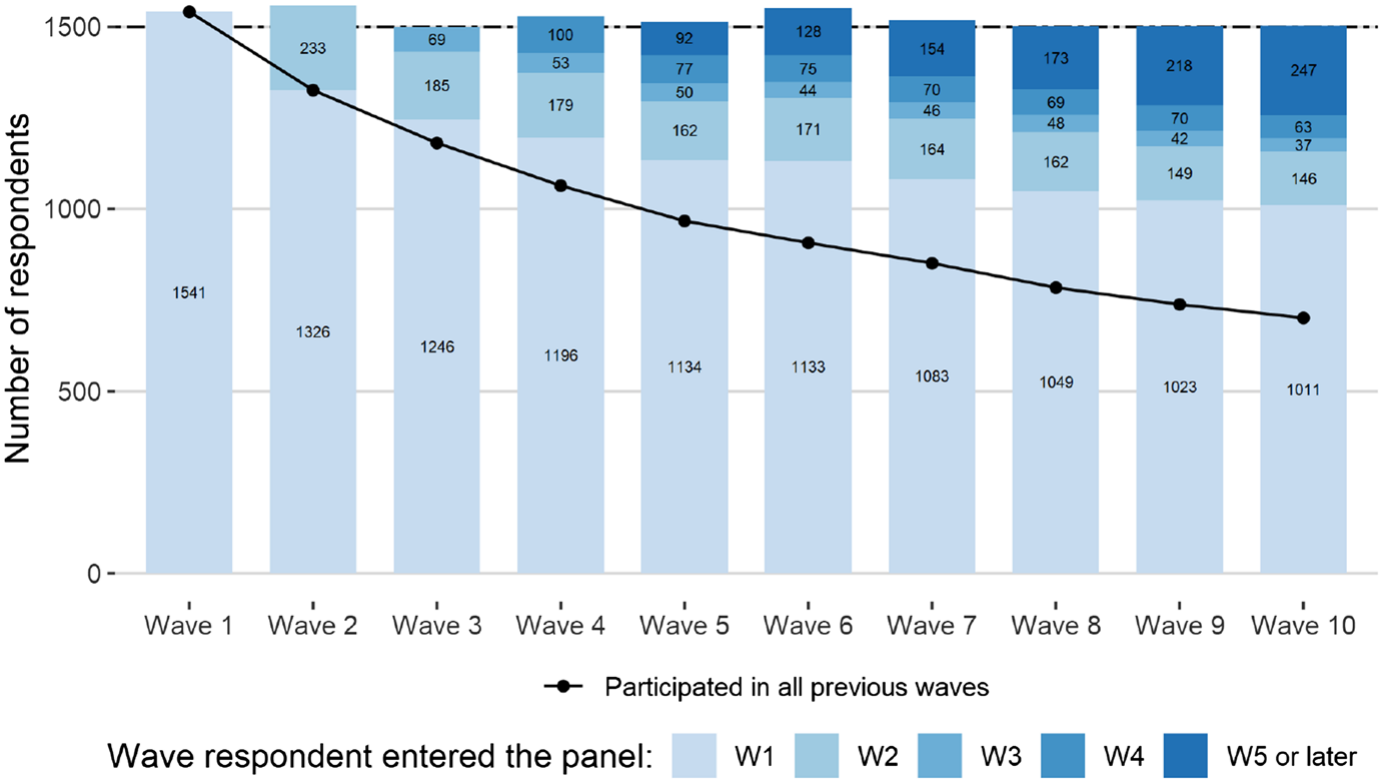
Composition of waves by panellists’ time of entry

Respondents received 180 credit points (1.80 €) for each wave in which they participated in the panel survey, which took about 19 min to complete on average. If the number of respondents was not sufficiently large, *n* ≤ 1500, new respondents were invited a few days after fieldwork started. Figure 2 shows that 1011 of the initial respondents (65.6%) from the first wave still took part in wave 10, with varying patterns of unit non-response. The second largest group in wave 10 consists of respondents recruited in the second wave (146), which emphasizes that we were able to motivate respondents to stay active. The black dotted line in Fig. 2 indicates the share of respondents in each wave that completed all previous questionnaires. While this share is clearly decreasing after each wave, still about half (45.5%) of wave-1-respondents (701) completed all ten waves.

The patterns of panel attrition are almost entirely unsystematic. To evaluate which respondents remained active, three regression models were estimated: one explaining which respondents took part in both wave 1 and wave 10, the second explaining which respondents took part in all ten waves, and the third explaining the number of waves a respondent participated in. Key demographics such as age, gender, age*gender, education, region, household size, employment status, and migration background were used as independent variables. As further predictors respondents’ perceived personal and public health-related and economic threats were included in the retention models (see Table 4 in “Appendix” for the full estimation results). Young respondents are less likely to stay active. In particular, young male respondents participated in fewer waves—a familiar pattern that can be found in the context of comparable studies (Aichholzer et al. 2020). Apart from that, only respondents from one regional entity (Burgenland) were somewhat less likely to be retained than others. Otherwise, there are no significant relationships with panel retention. Overall, the analyses show that there are hardly any systematic patterns, implying that panel drop-out has been essentially unrelated to key demographics and perceived COVID-19 threats.

## Research potential

While in the early days of the lockdown the focus of public debate was on individual behaviour and the reduction of infection risk, it soon became clear that the crisis could have lasting and disruptive effects on social life, the economy as well as political views and behaviours. The data enable researchers to study not only general developments like citizens’ perceptions of the pandemic, but also how respective policy measures affected behaviour and psychosocial dynamics within individuals and the society at large in a variety of issues.

The multidisciplinary approach of ACPP engenders a broad and varied palette of variables, which are included in the questionnaire and can be grouped into six main themes: health, economic situation, social situation, psychological conditions, political attitudes, and media and information. Table 2 illustrates the research potential of the data set, indicating some of the modules of the survey and specifying the waves in which they were included in the questionnaire.^4^ As noted, a core set of variables focusing especially on the immediate impact of COVID-19 on, for instance, employment situations and infection hazards has been asked weekly from wave 1 to wave 10. Many additional items were included in at least two waves and answered by the same respondents so that developments over time cannot only be traced at the aggregate level but also at the individual level. As a result, the data offer a unique possibility to analyse individual and societal short-term responses to crisis dynamics and political pandemic responses and the longer-term evolution of attitudes and behaviours in parallel.

**Table 2:** Module overview by panel wave (selection)

Theme	Module	1	2	3	4	5	6	7	8	9	10
General	Basic socio-demographics	✓	✓	✓	✓	✓	✓	✓	✓	✓	✓
	Crisis perception	✓	✓	✓	✓	✓	✓	✓	✓	✓	✓
Health	COVID-19 and risk of infection	✓	✓	✓	✓	✓	✓	✓	✓	✓	✓
	Legal drug use		✓		✓		✓		✓		✓
	Sleeping					✓			✓		✓
	Vaccination									✓	
Social situation	Latent deprivation and childcare	✓	✓	✓	✓	✓	✓	✓	✓	✓	✓
	Moving pattern and frequency	✓	✓	✓	✓	✓	✓	✓	✓	✓	✓
	Social norms and behaviour	✓	✓	✓				✓			
	Time use and gendered division of labour		✓			✓			✓		
	Community and solidarity			✓			✓			✓	
	Distributive justice norms					✓				✓	
	Daily routines and occupational balance						✓			✓	
	Perspectives after crisis			✓			✓				
	Elderly care		✓			✓					
	Home schooling						✓				
	Conflict in the household					✓					
	Values					✓					
	Private and public transport									✓	
Economic situation	Work situation and expectations	✓	✓	✓	✓	✓	✓	✓	✓	✓	✓
	Financial satisfaction	✓	✓	✓	✓	✓	✓	✓	✓	✓	✓
	Holiday						✓				
	Consumption								✓		
Psychological conditions	Emotions	✓	✓	✓	✓	✓	✓	✓	✓	✓	✓
	Life satisfaction	✓	✓	✓	✓	✓	✓	✓	✓	✓	✓
	Risk attitude and locus of control	✓					✓				
	Coping					✓				✓	
Political attitudes	Governmental measures	✓	✓	✓	✓	✓	✓	✓	✓	✓	✓
	Trust in institutions	✓	✓	✓	✓	✓	✓	✓	✓	✓	✓
	Satisfaction with democracy	✓	✓	✓	✓	✓	✓	✓	✓	✓	✓
	Populism and democratic preferences			✓		✓	✓			✓	
	Satisfaction with the government			✓		✓			✓		✓
	Surveillance measures	✓	✓		✓						
	Taxation				✓						✓
	Technology, robotic, and AI						✓	✓			
	Social policy and welfare attitudes								✓	✓	
	European Union		✓					✓			
	Climate change									✓	
	Migration									✓	
	E-Voting					✓					
Media and information	Corona concern	✓	✓	✓	✓	✓	✓	✓	✓	✓	✓
	Traditional and social media use	✓	✓						✓		
	Corona fake news		✓							✓	
	News avoidance and effects			✓						✓	
	Role of media			✓							
	Trust in media			✓							
	Stop-Corona App				✓						
	Perceived incivility									✓	

In the following section, we illustrate the research potential of ACPP by outlining some key developments during the crisis. After investigating respondents’ subjective threat perceptions, we will focus on some of the main topics already outlined in the introduction: economic situation, psychosocial conditions, political attitudes, and information and communication habits.

### Perceptions: threats

Perceptions are crucial to understanding the links between macro-level conditions and micro-level behavioural as well as attitudinal responses (Hedström and Swedberg 1998). Actual COVID-19 infection risks or unemployment risks may not straightforwardly translate into threat perceptions but depend on various moderating factors. Recent studies already provide first insights that political affiliation and media consumption (Barrios and Hochberg 2020), as well as trust in government, are linked to COVID-19 threat perception (Dryhurst et al. 2020). In a more theoretical sense, Schwarzer argues that perceptions “set[s] the stage for a contemplation process and further elaboration of thoughts about consequences and competencies” (Schwarzer 2008: 6). Hence, focusing on respondents’ perceptions should provide us with an idea of how the crisis unfolded from a subjective perspective and enable a better understanding of the attitudinal and behavioural responses that followed.

**Fig. 3:**
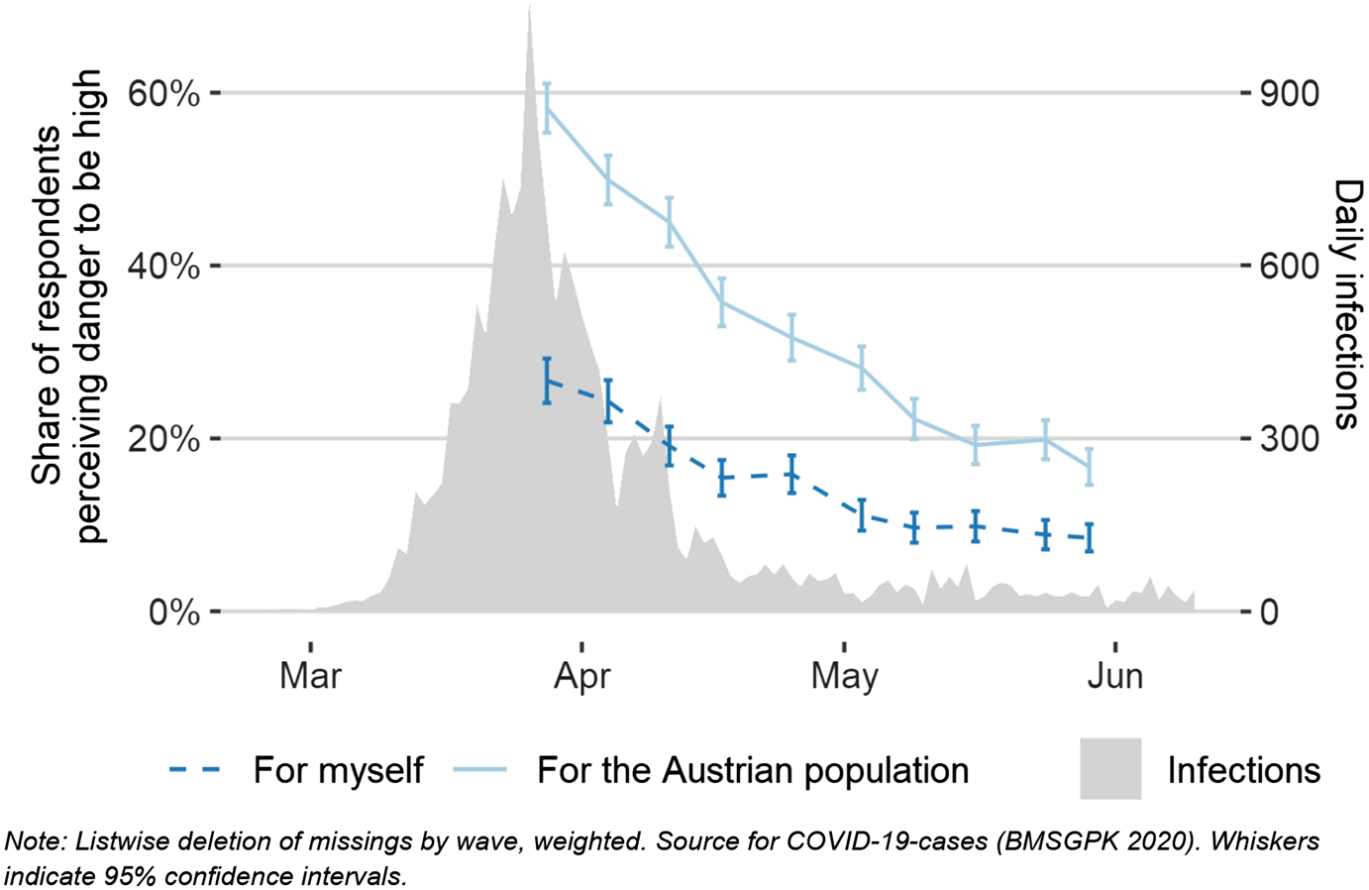
Health threat perception on a personal and a wider public level, combined with official reports of daily infections with the Coronavirus

**Fig. 4:**
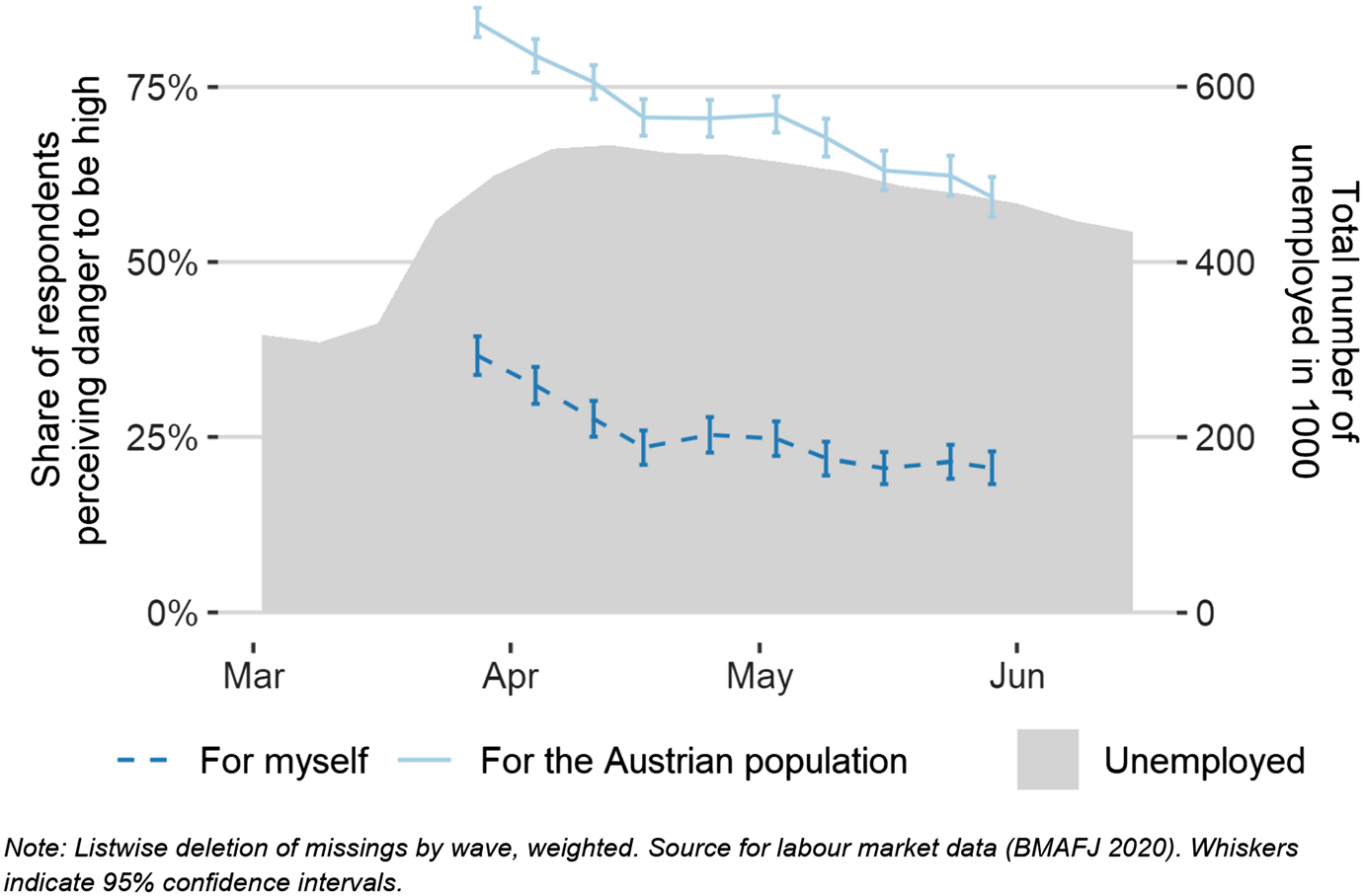
Economic threat perception on a personal and a public level, combined with weekly official reports on the total number of unemployed in Austria

The share of respondents in the ACPP who perceived health threats reached its high peak already in the first wave of the survey in late March 2020 (Fig. 3). Since then, threat levels have been decreasing and have somewhat levelled out by the mid of May 2020. These results coincide with the number of reported new cases of COVID-19 infections in Austria, although the latter number has dropped faster than the corresponding threat perception. In line with findings from earlier research on health-related risk perception in Great Britain (Rudisill 2013), we found that a substantially larger share of respondents perceived the threat that COVID-19 posed to the wider public to be higher than the personal threat. This might be an effect of the so-called optimism bias (Sharot 2011). This bias can also be found for respondents’ perceived economic threats (Fig. 4), which is again in line with the literature suggesting that people assign higher probabilities to occurrences of bad events to people in general compared to themselves (Boomgaarden et al. 2011; Rehm 2016). The differences between perceived personal and public economic threats of COVID-19 are even more pronounced than in the domain of health-related threats. However, again they seem to follow a similar decreasing pattern: It is noteworthy that perceived economic threats were already decreasing as unemployment rates were still increasing, suggesting that the economic expectations were extremely unfavourable at the height of the crisis.

### Crisis impact: employment conditions

**Fig. 5:**
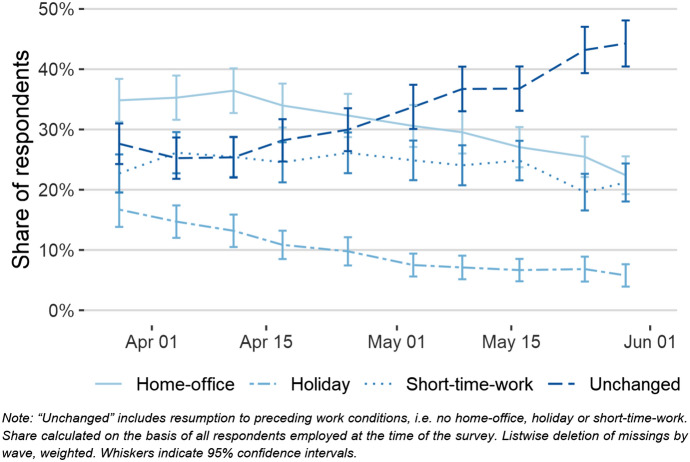
Employment conditions change over time

One explanatory factor of perceived economic threats might be the actual experience of changes in employment status or in employment conditions. As many businesses had to close to comply with governmental measures to curb the pandemic, many people faced severe changes in their employment status or conditions. This can also be seen in aggregate data as the crisis led to a pronounced increase in the unemployment rate in Austria from 8% in February to the crisis maxima of 13% in April (BMAFJ 2020). Those who kept their jobs, however, had to deal with changes as well: At the beginning of June 2020, still 1.2 million people in Austria were in short-time work, a governmental programme that allows a temporary reduction in working hours while maintaining the employment relationship and granting almost full public wage compensation. Among those who were not affected by changes in employment status or in employment conditions, many had to take annual leave or consume compensatory time, and many were ordered to work from home to meet physical distancing rules. In our data, the change of these statuses over time illustrates the course of the crisis (Fig. 5).

The consumption of annual leave and compensatory time was high in the beginning of the crisis but declined quickly, as employers could only oblige employees to take annual leave days under certain preconditions, such as the consumption of only 2 weeks of vacation from the current year.^5^ The share of short-time work remained at a similar level over the whole investigation period, dropping to a little more than 20% by the beginning of June 2020. The proportion of people working from home has decreased steadily as well: By June 2020, 45% of the respondents had returned to their usual work practices. The share of employees in our sample who reported at least one change of their employment status or condition during the crisis reached the mark of 70% in the last wave, indicating the immense impact of the crisis on employment. This also highlights the large variation in work conditions that people faced in this short period of time, which will allow us to fully utilize the potentials of panel data to assess causal effects. With the short period between waves and the stark contrasts in conditions, the ACPP research design enables researchers to closely investigate potential mechanisms.

### Emotional responses: loneliness

Throughout the pandemic, social life was heavily impacted by the measures imposed to control it: The restrictions on movement entailed that many could not see their families and friends. As sociability is one of the fundamental needs of humans that cannot be substituted (Cacioppo and Patrick 2008), we explored the emotional responses to these constraints in our survey, attempting to measure the degree of depression among the respondents. The 9-item battery of emotions can be used to build an index of well-being, and such indices have shown to be highly valid as a screening instrument for depressivity (Krieger et al. 2014).

**Fig. 6:**
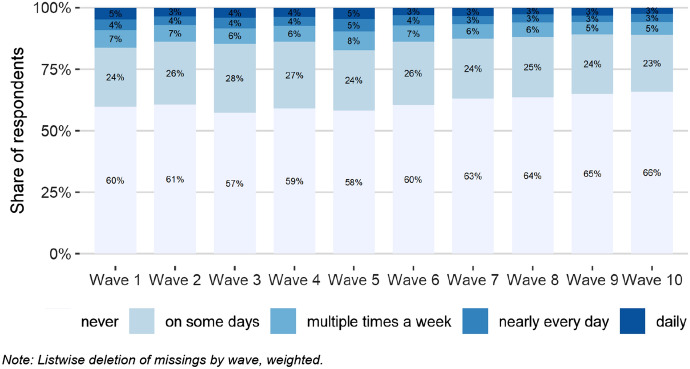
Distribution of loneliness in Austria during the COVID-19 crisis

One of the assessed emotions was the feeling of loneliness. The share of respondents feeling lonely several times a week or more frequently was 16% by the end of March. In the following weeks, this share declined to 14% in mid-April but rose again to 17% by the end of April 2020. Ever since, the share of persons having feelings of loneliness has been decreasing to 11% by early June 2020 (Fig. 6). While loneliness varies only in a limited way when considering the entire sample, some groups, such as unemployed people and students, had been affected more severely than others. This might be caused by fewer occasions to socialize compared to the pre-Corona situation and has persisted even after many measures had been lifted. The lonelier people feel, the less they tend to adhere to self-isolating norms, but show increased orientation on the behaviour of others instead. Through the closely knit assessment of emotions in our data, researchers can gain better understanding of the role of emotions in the crisis as well as disclose interrelations with behaviour.

### Behavioural responses: self-isolation

**Fig. 7:**
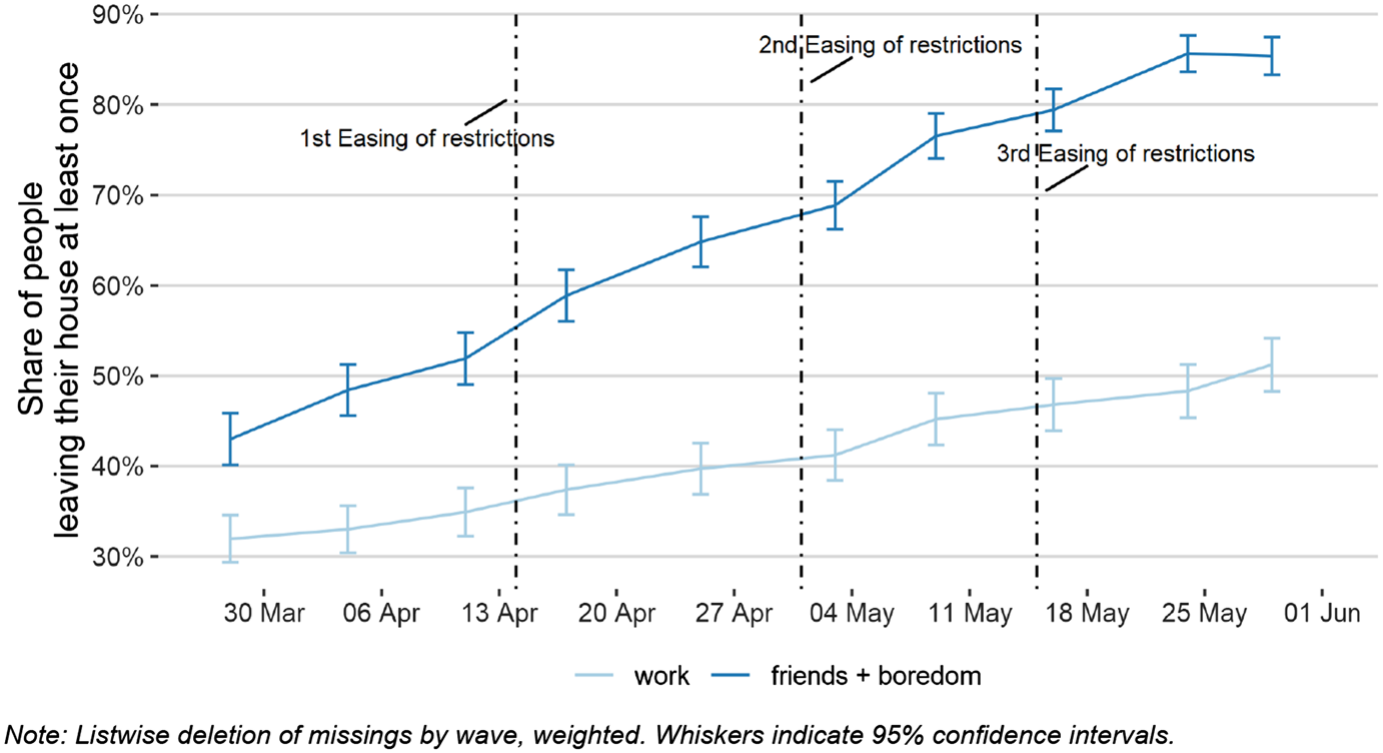
Reasons for leaving the house or flat, including dates of the lifting of restrictions

While perceptions and preferences are important to understand the reference points and possible motivators of individual decisions, behaviour is a tangible measure for the successful implementation of pandemic containment. To address this aspect in a survey context, we asked respondents to specify how often they left their homes for different reasons: As Fig. 7 shows, the respondents reported that self-isolation was high in the early phase of the crisis but had been declining ever since. This result corroborates findings from the analysis of geolocation data from mobile phones (cf. CSH Vienna and TU Wien 2020) and other tools to analyse moving patterns. However, our data provide even more detail: as respondents were asked to give specific reasons for leaving their home, we found significant differences between work and leisure activities as motivations to leave one’s home. Our results show that fewer people left their homes to go to work than to pursue leisure activities such as sports, meeting friends, or out of boredom.

Generally, the frequency of leaving the house has been steadily increasing since the beginning of our panel survey. As can be seen in Fig. 7, considerable kinks occur in the curve around each easing of governmental restrictions. This shows a slow boost of out-of-house activities by the regained possibilities to go shopping and visit bars and restaurants. However, further in-depth analyses are necessary to study how the frequency of leaving the house is linked to easings of certain governmental restrictions. Besides these aspects, research may investigate the relationship between self-isolating behaviour and personal or public threat perception. Additionally, the panel survey includes several questions on respondents’ own behaviour, their perceptions about the socially expected behaviour, and their perceived behaviour of others. This will enable in-depth research on the role of social norms, the importance of which has been pointed out before (van Bavel et al. 2020), as well as in compliance behaviour in the COVID-19 setting.

### Attitudes: government performance, solidarity, crisis expectations, and consumer sentiment

**Fig. 8:**
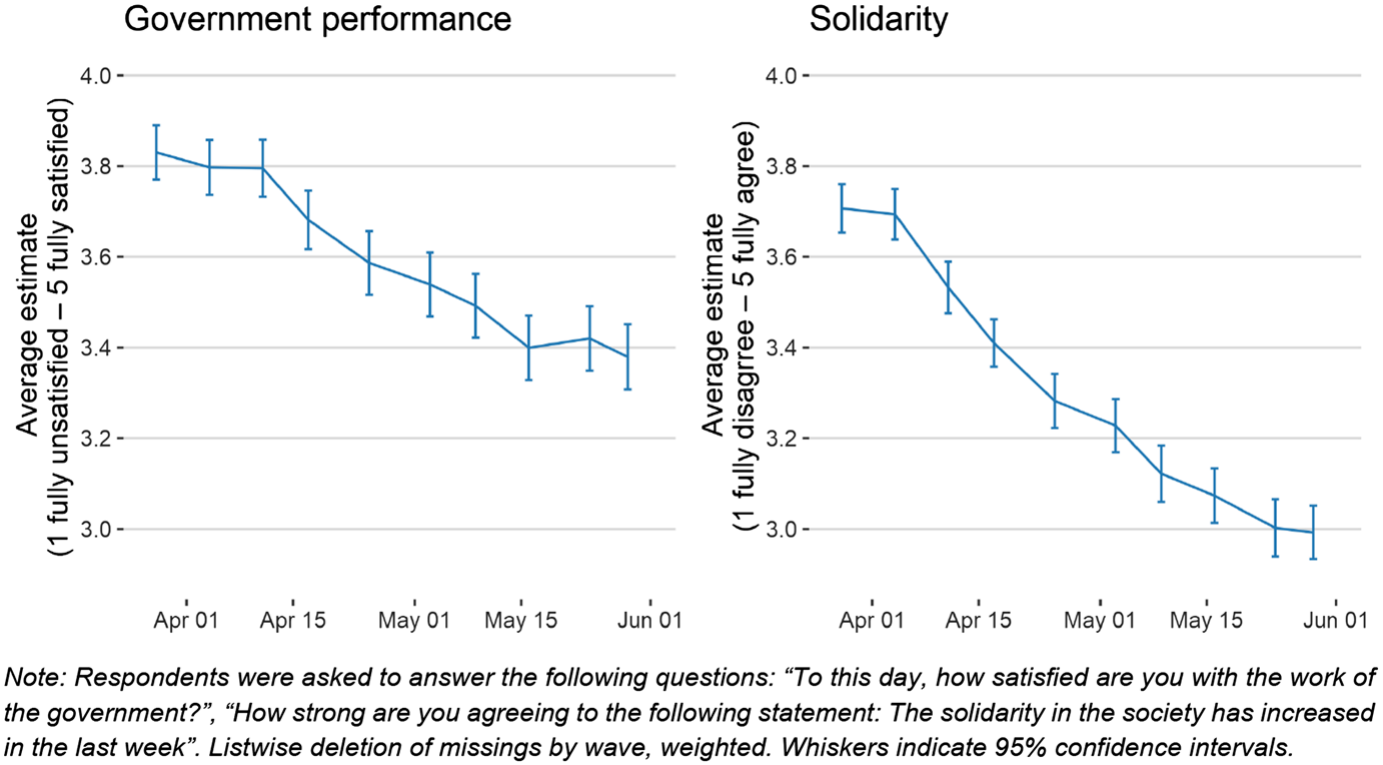
Satisfaction with government performance and perception of solidarity in Austrian society

Political scientists highlighted the large increase in incumbent satisfaction and voting immediately after the crisis (De Vries et al. 2020). This effect (Oneal and Bryan 1995) can also be seen in our data. At the height of the crisis at the end of March 2020, respondents’ positive evaluations of governmental performance reached a maximum and decreased steadily thereafter. But, while a considerable amount of literature has been published on rally-around-the-flag effects concerning the COVID-19 situation (Bækgaard et al. 2020, Leininger and Schaub 2020, Devine et al. 2020), its causes are still poorly understood. Figure 8 reveals not only high satisfaction with governmental performance but also high levels of perceived public solidarity. Further research might investigate whether both are connected or interdependent, and whether there are implications on both personal behaviour and the perception of other people’s behaviour.

**Fig. 9:**
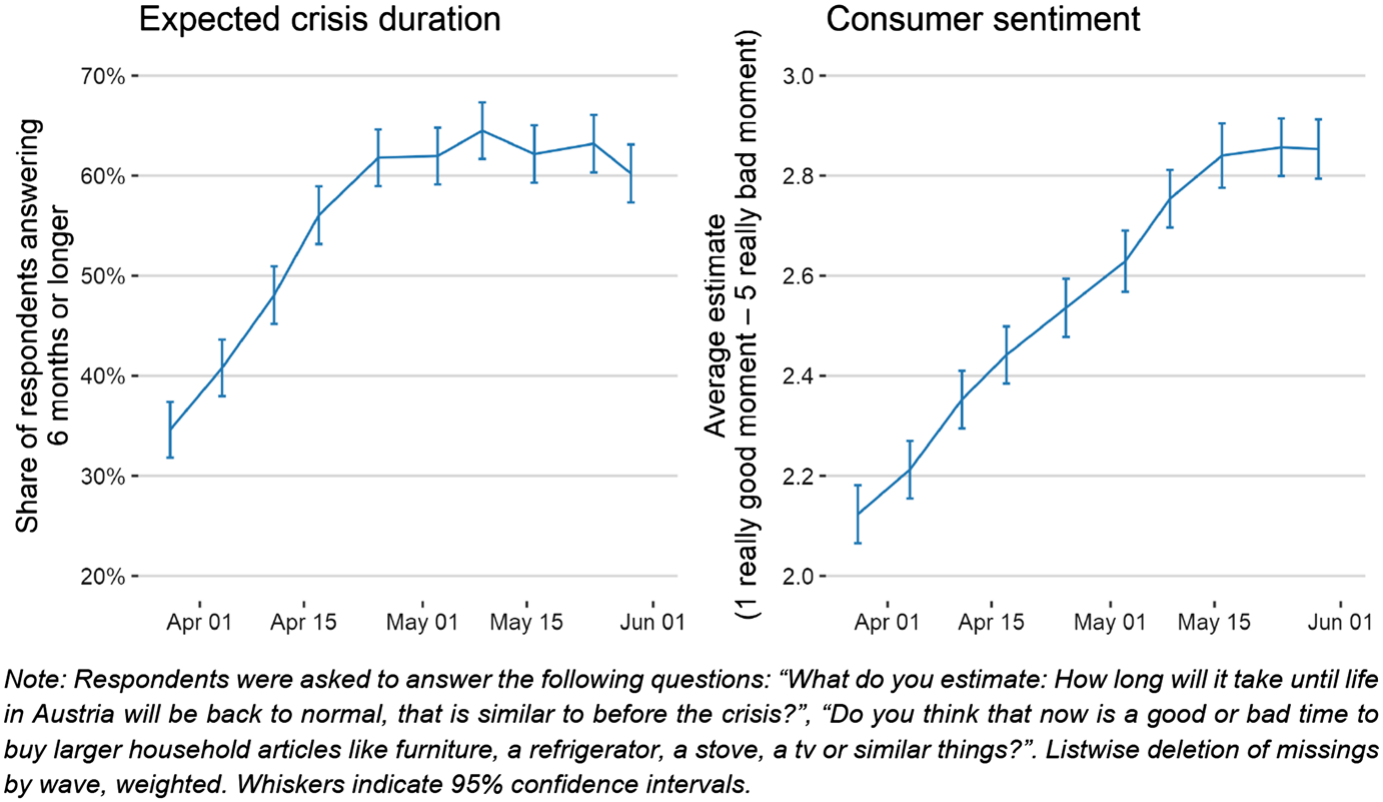
Share of respondents anticipating the crisis to last another 6 months or longer, and consumer sentiment

Over time, respondents’ estimation of the remaining duration of the COVID-19-crisis has notably risen: End of March 2020, a share of approximately 35% anticipated another 6 months of crisis or more. Until the end of April, this proportion had risen above 60%, staying at this level until June 2020. Alongside, consumer sentiment has consistently grown since the first wave of our panel survey, only levelling out since the end of May 2020 (Fig. 9). Hence, notwithstanding significantly increased expectations about the crisis’ duration, people do not want to postpone major acquisitions anymore. This coincides with a slow return towards “normal” working conditions and declining economic threat perceptions. Our data allow researchers to explore potential relations to many other aspects, like emotions, self-isolation, or applied coping strategies.

### Situational preferences: acceptance of governmental measures

**Fig. 10:**
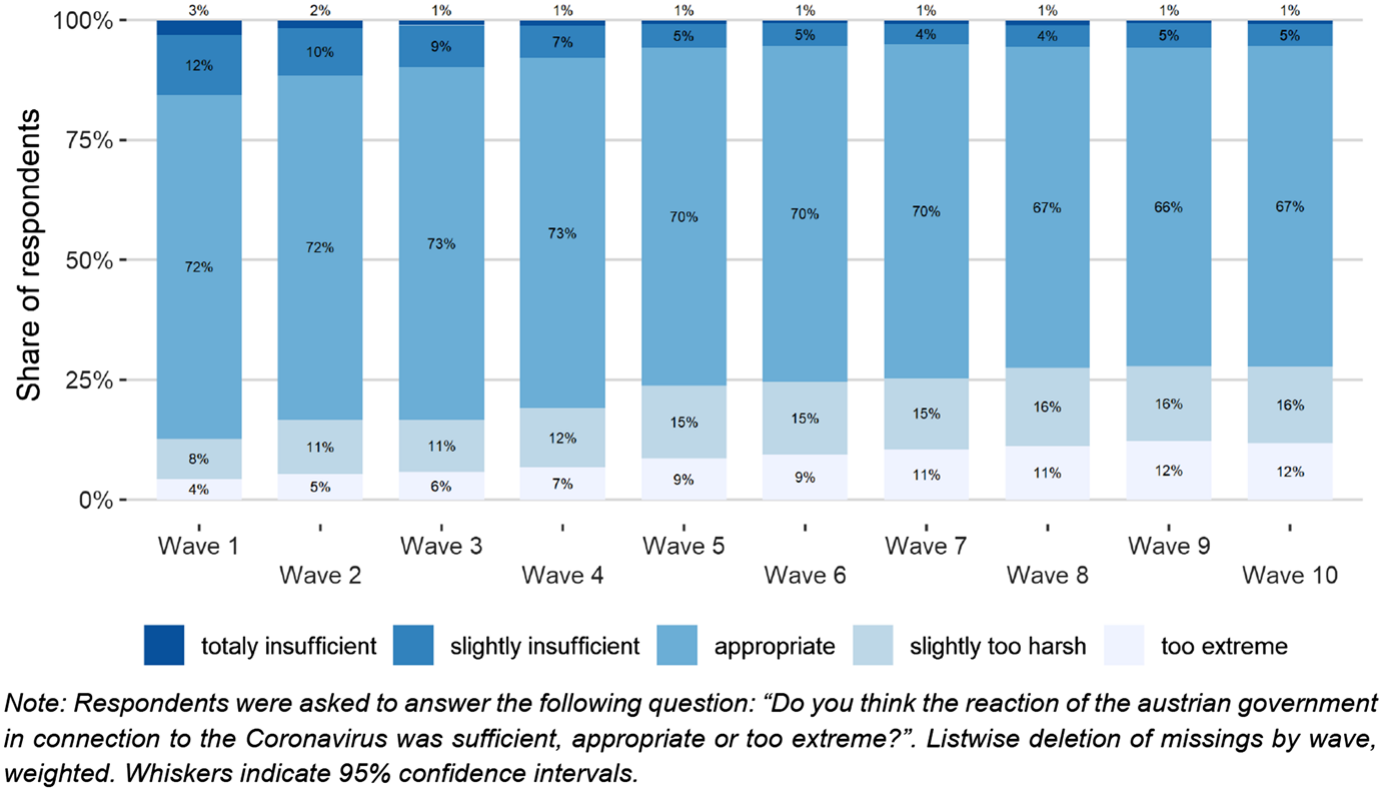
Acceptance of Governmental Measures

From the beginning of the crisis, the political measures to contain the spread of the coronavirus have been subject to public debate. Nonetheless, the share of respondents who thought that the imposed pandemic containment measures were adequate has been consistently high during the entire crisis, reaching levels between 73 and 66%. Still, we registered changes in the public approval of the measures: While the share of those who would have preferred even stricter regulations slightly exceeded the share of those who wanted to see the measures eased by the end of March 2020, this ratio was reversed soon. By the beginning of June, 28% of the respondents assessed the measures “rather too strong” or “too extreme” (Fig. 10).

The effectiveness of the measures has been perceived to be very high as well, with disagreement not exceeding 5–8 percent in all waves. Taking a look at the respondents’ agreement to certain measures, the opinions seem to follow official statements and the governmental schedule: Generally, the approval to all individual measures has been constantly decreasing since they were introduced. But the longer a measure had been in effect, the higher approval remained for the respective measure. With our data, researchers can examine whether or not this is related to rally-around-the-flag effects, and how possible interconnections with solidarity and perceptions of other people’s behaviour might look like.

### Communication: media use and COVID-19 concern

Media coverage enables broad sections of the population to keep informed about events that take place outside their own world of experience. This is particularly important in times of the COVID-19 pandemic, as the relevance of media information has increased even more due to social distancing, lockdown, and stay at home measures.

But not all citizens get their news from the same sources and particularly during the COVID-19 pandemic international organizations such as the WHO warned of an “infodemic associated with the current pandemic, i.e. the uncontrolled spread of false or misleading information about the virus, which might lead to different levels of COVID-19 concern in the population” (WHO 2020). In such instances international research points to professional journalism and especially a strong public broadcasting service as an essential corrective (Egelhofer et al. 2020; Humprecht et al. 2020).

**Fig. 11:**
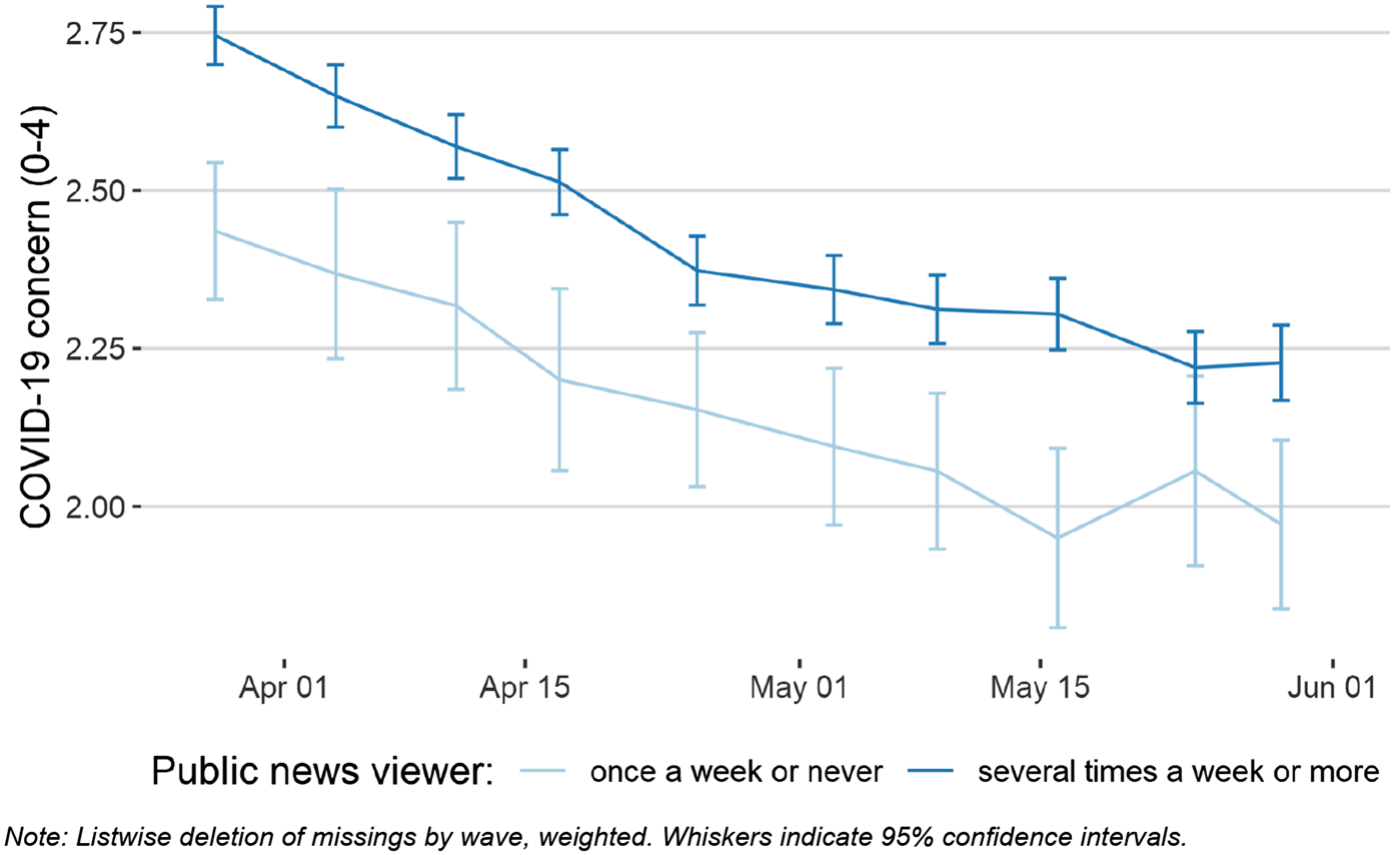
COVID-19 concern and public broadcasting news exposure

In fact, during the first weeks of the crisis, up to almost 80% of the respondents obtained their COVID-19 information more than once a week via the public broadcaster ORF. In Fig. 11, public news exposure was calculated using responses of the first two waves of the data. While the graph does not control for varying news consumption over the period of analysis and represents a simplified model of media effects, it shows clear differences in the level of COVID-19 concern.^6^ In accordance with the aforementioned theory, respondents regularly exposed to news from the public broadcaster were significantly more concerned by the developments of the crisis than those who refused that source of information.

## Data access

The Scientific Use File (SUF edition) (Kittel et al. 2020a) of the ACPP data covering the first ten survey waves is available for researchers and students from around the globe via download after registration at the Austrian Social Science Data Archive—AUSSDA (www.aussda.at). In addition, we provide a Public Use File (OA edition—open access edition) (Kittel et al. 2020b) that is suitable for the use by the interested general public that is also available via download from AUSSDA. Data from the ACPP are available in various data formats, including standard data formats such as Stata and SPSS and come together with a methods report and all questionnaires. ACPP is currently continuing data collection on a monthly rhythm. Data from subsequent waves will be released at regular intervals from autumn 2020 via AUSSDA.

## Conclusion

The ACPP data present a unique opportunity to study the effect of an exogenous shock that brought social and economic life to a near-halt in Austrian society. Being a country in which the government has implemented pandemic response fast and hard, followed by loosening measures after a few weeks, Austria offers an exemplary case to study the individual and societal dynamics during a crisis. The weekly individual-level panel structure of this publicly accessible data set provides vast opportunities to explore a variety of causal relationships and allows for most rigorous tests of theory-driven hypotheses. We have described some basic features of the data set and illustrated some potentials for more fine-grained and in-depth analyses in political science, sociology, communication science, economics, social psychology, and public health, among other disciplines. Supporting open science in the digital age, ACPP is looking forward to its data to be used widely and extensively.

## Appendix: The Austrian Corona Panel Project—monitoring societal dynamics amidst the COVID-19 crisis

**Table 3:** Distributions of key demographics

	Target	W1	W2	W3	W4	W5	W6	W7	W8	W9	W10
Male	48.8	49.7	49.4	49.3	49.9	49.2	49.7	49.6	49.7	49.3	50.1
Female	51.2	50.3	50.6	50.7	50.1	50.8	50.3	50.4	50.3	50.7	49.9
15–24 years	12.5	14.0	13.5	13.3	13.6	13.2	13.0	12.5	12.7	13.0	13.6
25–34 years	15.9	15.3	14.9	15.7	15.2	16.1	16.1	16.4	17.2	15.9	15.7
35–44 years	15.6	18.0	17.6	17.7	17.4	17.9	18.6	17.7	17.9	17.7	17.6
45–54 years	18.0	18.2	18.6	17.3	18.0	18.3	17.4	17.7	17.6	18.4	17.4
55–64 years	16.3	18.9	18.1	18.7	18.7	17.8	18.4	18.5	17.7	17.8	18.6
65 years+	21.6	15.5	17.3	17.2	17.1	16.8	16.6	17.3	16.9	17.2	17.2
Male, 15–24 years	6.4	6.6	6.0	6.0	6.1	6.1	5.7	5.2	5.5	5.7	6.2
Male, 25–34 years	8.1	7.2	7.5	7.8	8.2	8.4	8.5	9.1	9.8	8.9	8.7
Male, 35–44 years	7.8	9.0	9.6	9.4	9.3	9.4	9.8	9.2	9.2	8.8	9.0
Male, 45–54 years	9.0	9.3	9.5	9.1	9.3	9.2	8.7	8.9	8.6	9.0	8.6
Male, 55–64 years	8.0	9.8	9.1	9.3	9.5	9.0	9.8	10.0	9.3	9.5	10.3
Male, 65 years+	9.5	7.8	7.7	7.6	7.5	7.1	7.1	7.2	7.4	7.5	7.3
Female, 15–24 years	6.1	7.4	7.4	7.2	7.4	7.1	7.2	7.2	7.2	7.2	7.3
Female, 25–34 years	7.9	8.0	7.3	7.8	6.9	7.6	7.4	7.2	7.3	7.0	6.9
Female, 35–44 years	7.8	9.1	8.1	8.4	8.2	8.6	8.8	8.5	8.8	9.0	8.6
Female, 45–54 years	9.0	8.9	9.1	8.2	8.7	9.1	8.7	8.7	8.9	9.4	8.7
Female, 55–64 years	8.3	9.3	9.0	9.5	9.3	8.8	8.7	8.6	8.5	8.4	8.4
Female, 65 years+	12.1	7.7	9.6	9.6	9.6	9.7	9.5	10.1	9.5	9.7	9.9
Elementary or lower secondary school	21.3	11.9	11.3	11.1	10.3	10.3	10.4	10.0	10.0	9.5	10.1
Vocational school	34.1	46.0	45.8	46.4	46.8	45.8	45.2	45.6	45.2	45.9	46.8
Polytechnic, BMS	12.4	11.1	11.0	11.3	11.1	11.2	11.4	11.2	11.5	11.7	11.2
Upper secondary school	16.6	18.4	18.9	18.2	18.7	19.5	20.4	20.2	20.3	20.0	19.5
University degree	15.6	12.6	13.0	12.9	13.0	13.2	12.6	13.0	12.9	12.9	12.4
Vorarlberg	4.4	3.7	3.3	3.8	3.6	4.5	4.4	4.4	4.2	4.3	4.1
Tyrol	8.5	8.4	8.1	8.1	8.6	8.6	8.5	8.1	8.3	8.5	8.8
Salzburg	6.2	6.7	6.2	6.1	6.4	5.9	5.9	6.4	6.5	6.0	6.2
Styria	14.2	13.8	13.1	14.3	13.5	13.9	14.0	13.8	13.5	13.5	14.2
Carinthia	6.4	7.1	6.3	6.3	6.0	6.5	6.1	6.3	6.5	6.5	6.3
Upper Austria	16.6	16.6	16.4	16.3	16.6	16.6	16.9	16.9	17.6	16.7	16.7
Lower Austria	19.0	18.8	19.3	19.5	19.2	18.5	19.5	19.3	19.1	19.6	19.4
Vienna	21.3	21.0	23.9	22.1	22.8	22.5	21.7	21.8	21.3	22.2	21.5
Burgenland	3.4	3.8	3.4	3.5	3.3	2.9	3.0	3.1	3.0	2.7	2.8
1 person	19.9	21.1	21.8	21.3	21.9	20.7	21.0	21.3	21.1	20.3	20.2
2 persons	31.6	36.3	35.5	36.0	35.7	36.1	36.2	36.1	35.6	37.2	36.9
3+ persons	48.5	42.6	42.7	42.7	42.3	43.2	42.8	42.6	43.4	42.5	42.9
Employed	54.4	55.5	54.4	55.4	55.5	55.3	55.7	55.6	56.0	55.7	55.4
Retired	26.7	24.7	26.7	26.1	26.2	25.1	25.1	25.6	25.0	25.6	25.8
Pupil/student	7.1	9.9	9.5	9.1	9.3	10.0	9.8	9.6	8.9	9.0	9.2
Others	11.7	9.9	9.3	9.4	9.1	9.7	9.4	9.3	10.0	9.7	9.5
Migration background: no	76.8	80.5	80.1	79.5	79.5	78.9	79.0	79.4	79.6	78.4	78.6
Migration background: yes	23.2	19.5	19.9	20.5	20.5	21.1	21.0	20.6	20.4	21.6	21.4

Entries in per cent (%). Target values are from the Austrian Micro Census (Statistik Austria 2019)

**Table 4:** Panel retention

	(1) Participation in wave and wave 10 (0/1)		(2) Participation in all ten waves (0/1)		(3) Number of waves participated (1–10)	
Male	0.153	(0.313)	0.302	(0.272)	0.274	(0.393)
15–24 years	− 0.924*	(0.422)	− 0.969*	(0.416)	− 1.243*	(0.572)
25–34 years	− 0.959*	(0.381)	− 0.619	(0.357)	− 1.249*	(0.509)
35–44 years	− 0.701	(0.371)	− 0.117	(0.341)	− 0.585	(0.492)
45–54 years	− 0.506	(0.360)	0.097	(0.327)	− 0.395	(0.473)
55–64 years	− 0.300	(0.311)	− 0.147	(0.281)	− 0.178	(0.408)
Male, 15–24 years	− 0.363	(0.429)	− 0.428	(0.430)	− 1.156*	(0.578)
Male, 25–34 years	0.272	(0.431)	0.152	(0.400)	0.538	(0.572)
Male, 35–44 years	0.021	(0.415)	− 0.398	(0.378)	0.006	(0.545)
Male, 45–54 years	− 0.149	(0.412)	− 0.316	(0.370)	0.055	(0.535)
Male, 55–64 years	0.306	(0.421)	0.082	(0.369)	0.234	(0.533)
Education: elementary/lower secondary school	− 0.085	(0.246)	− 0.319	(0.241)	− 0.519	(0.339)
Education: vocational school	0.070	(0.188)	0.039	(0.178)	0.145	(0.256)
Education: polytechnic, BMS	0.223	(0.247)	0.126	(0.230)	0.152	(0.329)
Education: upper secondary school	− 0.154	(0.206)	− 0.272	(0.201)	− 0.017	(0.285)
Region: Vorarlberg	0.182	(0.346)	− 0.379	(0.312)	0.146	(0.436)
Region: Tyrol	− 0.273	(0.239)	− 0.053	(0.228)	− 0.347	(0.324)
Region: Salzburg	− 0.271	(0.250)	− 0.136	(0.240)	− 0.342	(0.341)
Region: Styria	− 0.039	(0.200)	0.004	(0.190)	− 0.068	(0.270)
Region: Carinthia	0.102	(0.253)	− 0.033	(0.236)	0.018	(0.334)
Region: Upper Austria	− 0.271	(0.187)	− 0.307	(0.180)	− 0.225	(0.255)
Region: Lower Austria	− 0.108	(0.187)	0.118	(0.176)	0.055	(0.252)
Region: Burgenland	− 0.869**	(0.309)	− 0.716*	(0.319)	− 1.259**	(0.432)
Household size: 1 person	− 0.112	(0.164)	0.003	(0.156)	− 0.052	(0.222)
Household size: 2 persons	− 0.066	(0.141)	− 0.021	(0.135)	− 0.001	(0.192)
Migration background: yes	0.066	(0.145)	0.204	(0.138)	0.382	(0.195)
Employment status: retired	− 0.183	(0.243)	− 0.102	(0.222)	0.106	(0.320)
Employment status: pupil/student	− 0.014	(0.255)	0.245	(0.278)	0.510	(0.367)
Employment status: others	0.084	(0.203)	0.168	(0.195)	0.312	(0.277)
Health risk: respondent	0.043	(0.064)	0.086	(0.061)	0.087	(0.086)
Health risk: Austrian population	− 0.068	(0.081)	− 0.039	(0.077)	− 0.097	(0.110)
Economic risk: respondent	0.068	(0.056)	0.030	(0.053)	0.040	(0.076)
Economic risk: Austrian population	− 0.018	(0.084)	0.032	(0.081)	0.056	(0.114)
Constant	1.361**	(0.472)	− 0.132	(0.438)	7.853***	(0.627)
*N*	1480		1480		1480	
*R*^2^					0.063	
Pseudo-*R*^2^	0.034		0.041			

Entries for models (1) and (2) are logit coefficients; for model (3), unstandardized regression coefficients

Standard errors in parentheses

**p* < 0.05, ***p* < 0.01, ****p* < 0.001

## Footnotes

1 An overview of other national (panel) data sets can be found for instance at https://lukaslehner.github.io/covid19policytrackers/#15-national-surveys-with-probability-samples.

2 Austria gradually reopened from the beginning of May, and had not entered another lockdown at the time of writing this article.

3 As part of the project, we released over 60 (mostly German but also English) scientific blog posts to numerous topics to present first results utilizing the ACPP data. The paragraph summarizes some of the results presented in these blogs that can be accessed at: https://viecer.univie.ac.at/coronapanel/corona-blog/.

4 Because of space restrictions, we had to considerably reduce the amount of detail of the modules in the survey containing nearly 800 variables. For a full overview, see the variable description offered alongside the data.

5 See 2nd COVID-19 law, BGBl. I Nr. 16/2020 (entered into force March 21, 2020). https://www.ris.bka.gv.at/eli/bgbl/I/2020/16.

6 COVID-19 concern was operationalized on the basis of the ISSP measure of “environmental concern” (see ISSP Environment III 2010—https://search.gesis.org/research_data/ZA5500) and calculated using the degree of disagreement to the following statements: “There are more important things in life than the coronavirus”, “Claims about the crisis are exaggerated”, “I am willing to restrict my lifestyle to contain the virus” (inverted), “As long as others do not change their behavior I do not have to change it either”.
